# The Effect of Pioglitazone on Antioxidant Levels and Renal Histopathology in Streptozotocin-Induced Diabetic Rats

**DOI:** 10.1155/2013/858690

**Published:** 2013-05-09

**Authors:** Munire Kuru Karabas, Mediha Ayhan, Engin Guney, Mukadder Serter, Ibrahim Meteoglu

**Affiliations:** ^1^Department of Internal Medicine, Manisa Government Hospital, 45000 Manisa, Turkey; ^2^Division of Endocrinology, Department of Internal Medicine, Adnan Menderes University Medical Faculty, 09100 Aydin, Turkey; ^3^Department of Medical Biochemistry, Adnan Menderes University Medical Faculty, 09100 Aydin, Turkey; ^4^Department of Pathology, Adnan Menderes University Medical Faculty, 09100 Aydin, Turkey

## Abstract

*Objective*. Diabetic nephropathy is the most commonly seen cause of chronic renal failure, and oxidative stress is important in etiology. In the present study, favorable effects (if any) of the treatment with a thiazolidinedione group drug, pioglitazone, on antioxidant enzyme levels in the renal tissue, renal histopathology, and inflammatory cytokine levels have been investigated. *Method*. Forty male Wistar rats were divided into 4 groups as the control, diabetic control, and 10 and 30 mg pioglitazone-administered diabetic groups. After 4 weeks, antioxidant enzyme levels in renal tissues and inflammatory markers were investigated. *Results*. Blood glucose levels did not differ between the diabetic control and drug-administered groups. In pioglitazone-administered rats, histopathological findings such as tubular dilation, necrotic tubular epithelium, glomerular focal necrosis, and vascular consolidation were observed at a lesser extent than the diabetic control group. Any difference was not detected between the diabetic groups with respect to the levels of malondialdehyde, superoxide dismutase, catalase, glutathione, nitric oxide, interleukin-6, and tumor necrosis factor-alpha. *Conclusion*. Pioglitazone regressed development of histopathological lesions such as glomerular focal necrosis, tubular epithelial necrosis, tubular dilation, and vascular wall consolidation. However, any favorable effect on antioxidant enzyme levels in renal tissues and inflammation markers was not detected.

## 1. Introduction

Diabetes mellitus has become the most frequently seen global etiological factor for the end-stage renal failure. According to the data published by World Health Organization, in the year 2030, the number of diabetics was predicted to amount to 370 million patients [1]. As proved in many investigations, strict glycemic and heart rate control prevent occurrence and progression of diabetic nephropathy [2–4]. However, especially in some of the type 2 DM patients, complications already develop at the time of diagnosis, and strict glycemic control cannot be always achieved. Therefore, development of treatment modalities preventing occurrence or progression of diabetic nephropathy seems to be an urgent need. Pathophysiological mechanisms contributing to the formation of diabetic nephropathy and treatment modalities directing to that end have been investigated. Increased activation of polyol, protein kinase c and hexosamine pathways, and intracellular AGEs (advanced glycation end products) were determined as basic mechanisms of hyperglycemic tissue damage [5]. Oxidative stress is the common denominator involved in all of these pathways. In hyperglycemic states, generation of free oxygen radicals is accelerated, and antioxidant defense systems are weakened [6–11]. 

Thiazolidinediones (TZDs) are insulin-sensitizing agents used in the treatment of type 2 diabetes. They demonstrate these effects by activating peroxisome proliferator activating receptor gamma (PPAR*γ*) which is a type of nuclear receptor and acts as a PPAR*γ* agonist [12]. PPAR*γ* receptors play important roles in differentiation of adipocytes, lipid, and carbohydrate metabolism via transcriptional regulation of various genes. Even though they are mostly found in adipose tissue, they are also detected in vascular smooth muscle cells, macrophages, vascular endothelial cells, colonic epithelial cells, and renal glomerular cells [13]. Favorable effects of TZDs on diabetic nephropathy have been reported [14–18]. 

 In the present study, irrespective of its antiglycemic effects, we planned to investigate the impact of pioglitazone on antioxidant enzyme levels in renal tissue and renal histopathology. 

## 2. Material and Method

Forty male Wistar rats weighing 200–250 g were used in this study. Rats were brought into a special room with a stable ambient temperature of 18°C–22°C, 10 days before the experiment. Rats were placed by fives in a cage and fed with a standard pellet diet. Rats were given water and pellets ad libitum. Blood glucose levels of all rats were measured before the experiment. 

 Rats were divided into four groups as the control group (*n* = 10), diabetic control group (*n* = 10), and diabetic groups which received 10 mg (*n* = 10) and 30 mg pioglitazone groups. In addition, streptozotocin was injected intraperitoneally to 30 rats at a dose of 50 mg/kg. Streptozotocin was dissolved in a sodium citrate buffer (1 mL/kg) solution. The remaining 10 rats, which consisted the control group, received i.p. citrate buffer injections. On the third day, glycemic measurements were performed in blood samples drawn from tail veins of rats to determine whether rats were diabetic or not. Glycemic levels were measured using a glucometer device Accu-chek Go (Roche Diagnostics, Meylan, France). Rats with blood glucose levels ≥250 mg/dL were considered as diabetic and included in the study. Two groups of diabetes-induced rats received pioglitazone (Glifix) (10 mg/kg and 30 mg/kg) mixed with their food. Rats in the other diabetic group were set apart as a diabetic control group. Four weeks later, the rats were nephrectomized under xylazine anesthesia. Right kidneys of the rats were reserved for histopathological examination, and left kidneys were taken apart for the assessment of biochemical parameters. Renal tissues were preserved under −80°C till the time of analysis. For this study, ethics committee approval was obtained from Adnan Menderes University, Ethics Committee for Animal Experiments.

### 2.1. Biochemical Measurements

*Tissue homogenization* was performed in Braun brand homogenisator using tissue homogenization buffer. Tissue homogenization buffer (1 mM, pH = 7.4) was prepared using phenylmethylsulfonylfluoride (C_7_H_7_FO_25_, SIGMA, Catalogue no. P-7626), di-natrium hydrogenphosphate-dihydrate (Na_2_HPO_4_·2H_2_O, MERCK, Catalogue no. K25979680), potassium dihydrogenphosphate (H_2_KPO_4_, MERCK, Catalogue no. A986373), and ethylenediaminetetraacetic acid disodium (EDTA), (C_10_H_14_N_2_O_8_Na_2_·2H_2_O, SIGMA, Catalogue no. E-1644). Renal tissue antioxidant levels were measured as follows.

*Tissue MDA levels* were assessed indirectly by the measurement of tissue TBARS (thiobarbituric acid reactive species) levels. Tissue analyses were performed in accordance with Draper and Hadley [19]. The standard solution was prepared using phosphoric acid (1%) (phosphoric acid MERCK 1.00563) and TBA (0.6%) (2-thiobarbutiric acid, 4.6-dihydroxypyrimidine-2-thiol, SIGMA, Catalogue no. T-5500). MDA standard was prepared using malonaldehyde bis (dimethyl acetal) (ALDRICH, AL-108383). Samples and standards were read on Shimadzu UV-160 A spectrophotometer and evaluated against a blind solution at 532 nm.

*Reduced GSH* was determined in accordance with Beutler et al. [20]. Precipitating solution was prepared using glacial metaphosphoric acid (RIEDEL-de HAEN 04103), disodium EDTA (C_10_H_14_N_2_O_8_Na_2_, 2H_2_O, SIGMA, Catalogue no. E-1644), and sodium chloride (NaCl, J.T. Baker). Disodium phosphate solution was prepared using disodium hydrogen phosphate (Na_2_HPO_4, _MERCK, Catalogue no. F368386). DTNB solution was formulated using DTNB 5,5′-dithio-bis(2-nıtrobenzoic acid) (C_14_H_8_N_2_O_8_S_2_, SIGMA, Catalogue no. D-8130) and sodium citrate (C_6_H_5_Na_3_O_7_·2H_2_O, SIGMA, Catalogue no. S-4641). Glutathion standard was prepared using Glutathione Reduced Form, (C_10_H_17_N_3_O_6_S, SIGMA, Catalogue no. G-4251) standards, and blind solutions were read on Shimadzu UV-160 A spectrophotometer against a blind solution at 412 nm.

*Tissue CAT activity* was determined in accordance with Hugo Aebi method [21]. Buffer solution (50 mM pH = 7) contained potassium dihydrogenphosphate (KH_2_PO_4_ MERCK, Catalogue no. A986373) and disodium hydrogen phosphate (Na_2_HPO_4_·2H_2_O, MERCK, Catalogue no. K25979680). Buffer solution with H_2_O_2_ was prepared by adding hydrogen peroxide (H_2_O_2_, RIEDEL, RH18312) to the already formulated buffer solution. Change in absorbance started with the addition of buffer isolation with H_2_O_2_ to the sample solution diluted with the buffer solution, and it was monitored for 15 seconds and determined at 240 nm on Shimadzu UV-160 A spectrophotometer. Using a relevant formula, the change in absorbance was calculated based on spectrophotometric data. 

### 2.2. Determination of Tissue NO Metabolite

The level of nitrate, which is one of the degradation products of NO, was estimated indirectly, so as, to form an opinion about NO levels. For this estimation, the method proposed by Cortas and Wakid was used [22]. According to this method, cadmium (FLUKA, Catalogue no. 20890) granules were used. Glycine-NaOH buffer was prepared using glycine (C_2_H_5_NO_2_, MERCK, Catalogue no. K23214990) and sodium hydroxide (NaOH, PROLABO, Catalogue no. EMB 45053) solutions. CuSO_4_ solution contained in glycine NaOH buffer was prepared using glycine, NaOH, and Cu sulphate (CuSO_4_·5 H_2_O, RIEDEL, RH12849-1). For the preparation of sulfanilamide hydrochloric acid (37%) (HCl, MERCK, Catalogue no. K24016914) and sulfanilamide (C_6_H_8_N_2_O_2_S, SIGMA, Catalogue no. S-9251) solutions were used. NED isolation (N-naphthyl ethylenediamine) was prepared from ethylenediamine dihydrochloride (ALDRICH 22,248) solution. Standard solutions were prepared using sodium nitrite (NaNO_2_, SIGMA, Catalogue no. S-3421). The sample and standard solutions were read on ELISA microplate reader at 540 nm.

For *determination of tissue SOD activity*, the method popularized by Sun et al. was used [23]. SOD assay reactant was prepared using 0.3 m*μ* xanthine solution (xanthine, SIGMA SIX7375), 0.6 m*μ* EDTA solution (EDTA, ALDRICH 31788), 150 *μ*mol NBT (SIGMA SIN6639), 400 mmol Na_2_CO_3_ solution (MERCK 1.06392), and BSA solution (bovine serum albumin, SIGMA SIA7906). For the preparation of xanthine oxidase solution, xanthine oxidase (SIGMA SIX4376) was dissolved in 2 mmol ammonium sulphate solution (ammonium sulfate, RIEDEL RH31119). Reactions in prepared samples were stopped with the addition of 0.8 mmol CuCl_2_ to the test media. Absorbance was measured using Shimadzu UV 160 A spectrophotometer at 560 nm wavelength.

*TNF*α* and IL-6* in serum and tissue samples were determined using an ELISA kit. 

### 2.3. Histopathological Analysis

Paraffin blocks were prepared from tissue samples fixated in 10% neutral buffered formalin solution after routine tissue monitorization process. From each tissue sample, 4 mm thick sections were obtained, and these tissue sections were stained with routine hematoxylin-eosin, and other histochemical dyes including Masson's Trachome, methenamine silver, and PAS-Alcian Blue dyes, and examined under light microscope. Renal tissue samples were examined as for parameters of glomerular sclerosis, glomerular focal necrosis, thickening, and dilation of Bowman capsule, degeneration, and necrosis of tubular epithelium, interstitial inflammation, induration of the vascular walls, and interstitial fibrosis. These parameters were assessed using semiquantitative scoring and morphometric measurements. Pathological lesions observed in renal tissue samples were defined as unaffected (−), moderately (+), and severely affected (++) 

### 2.4. Statistical Analysis

For statistical evaluation of data obtained in our study, *chi-*square test, Kruskal-Wallis, and Mann-Whitney *U* tests were used. For the assessment of the difference between histopathological examination data of the kidneys, *chi*-square test was used. For the assessment of the difference between mean values of antioxidant and inflammatory parameters of the renal tissue, Kruskal-Wallis and Mann-Whitney *U* tests were performed. *P* < 0.05 was considered as a statistically significant cut-off value during assessments. For statistical evaluation of the study results, SPPSS 14 program was utilized. 

## 3. Results

### 3.1. Comparisons between Blood Glucose Levels

Blood glucose levels of rats at beginning and fourth week of the treatment were measured. Mean blood glucose levels in the diabetic control group were significantly higher than the corresponding mean values of the control group (*P* = 0.001). However, a significant difference between diabetic rats which were on drug therapy or not was not detected (Tables 1 and 2).

### 3.2. Antioxidants and Inflammatory Markers

A statistically significant difference was detected between control and diabetic control groups as for mean MDA values (*P* = 0.011). Significantly higher levels of MDA (a marker of lipid peroxidation) were detected in diabetic rats. However, with respect to mean MDA values, a significant difference between the diabetic control group and the pioglitazone groups was not detected. Similarly, a statistically significant difference was not found among mean SOD, CAT, GSH, NO, TNF-*α*, and IL-6 values of diabetic control, and pioglitazone groups was not found (Table 3).

### 3.3. Histopathological Examination Results of Renal Tissue Samples

**Figure 1:**
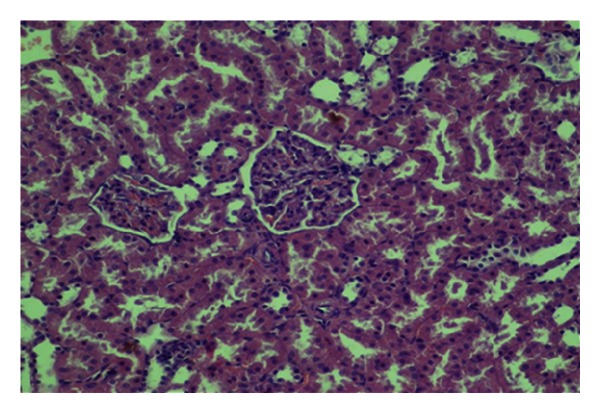
Mild congestionin renal tissue (HE, ×200).

**Figure 2:**
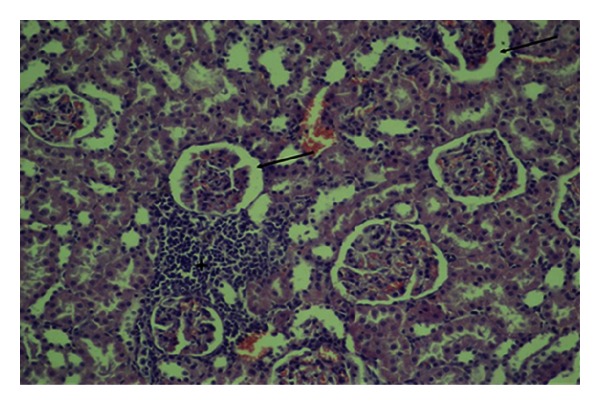
Congestion, interstitial inflammation (asterix), and dilation of Bowman capsular space (arrows) (HE, ×200).

**Figure 3:**
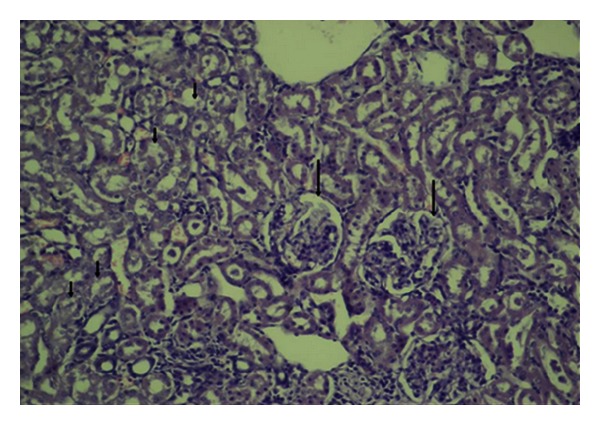
Tubular dilation, degeneration (short arrows), and focal glomerular necrosis (HE, ×200).

**Figure 4:**
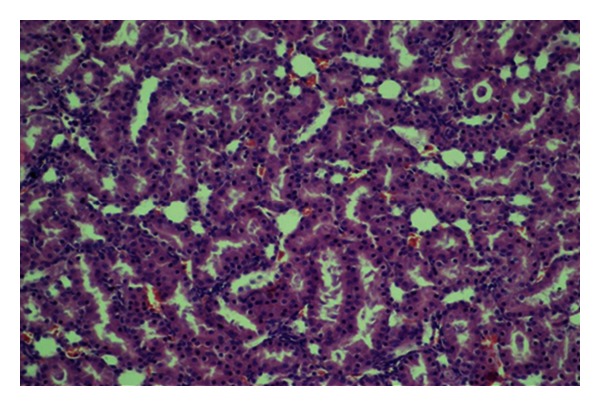
Physiological structure of renal tubuli (HE, ×200).

**Figure 5:**
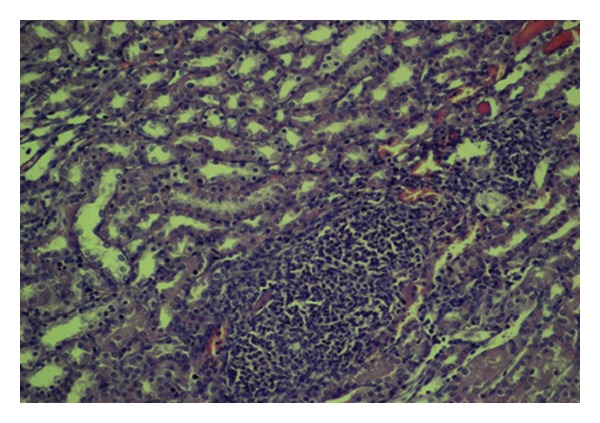
Diffuse interstitial inflammation, tubular degeneration, and dilation (HE, ×200).

**Figure 6:**
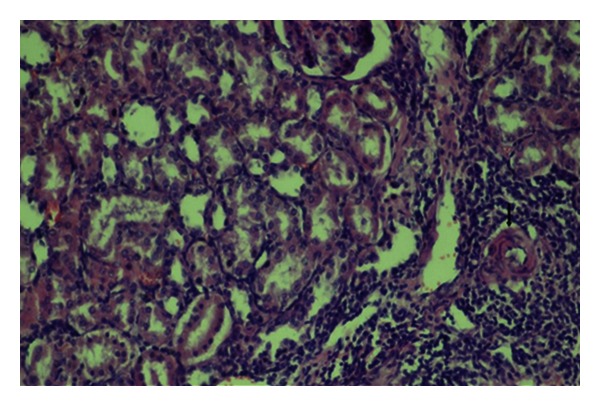
Interstitial inflammation, tubular degeneration, tubular dilation, and vascular wall thickening (arrow) (HE, ×200).

A statistically significant difference was detected between control and diabetic control groups with respect to glomerular focal necrosis, tubular dilation, and consolidation of the vascular wall (*P* = 0.033, *P* = 0.013, and *P* = 0.003, resp.) (Figures 1, 2, 3, 4, 5, and 6). A statistically significant difference was detected between diabetic control group and 10 mg pioglitazone group as for tubular epithelial necrosis, thickening of the vascular wall, and glomerular focal necrosis (*P* = 0.040, *P* = 0.007, and *P* = 0.031, resp.). Severe glomerular focal necrosis was seen in 57% of the rats in the diabetic control group; however, in the 10 mg pioglitazone group, glomerular focal necrosis was not so severe. Tubular epithelial necrosis was not observed in 14.3 and 66.7% of the rats in the diabetic control and 10 mg pioglitazone groups, respectively. Severe vascular wall consolidation was seen in 85.7 and only 11% of the rats in the diabetic control and 10 mg pioglitazone groups, respectively.

A significant difference was detected between the diabetic control and 30 mg pioglitazone groups as for tubular dilation, and vascular wall thickening. (*P* = 0.027 and *P* = 0.008, resp.). In the diabetic control group, severe tubular dilation and vascular wall thickening were observed in the diabetic control group (71.4 and 85.7%, resp.); these lesions were of mild degree in the diabetic group which received 30 mg pioglitazone. Even though a statistically significant difference was not detected between the diabetic control group and 30 mg pioglitazone group as for glomerular focal necrosis, in the diabetic control group lesions of 57% of the rats were more severe than those in the diabetic group, which received 30 mg pioglitazone. Besides, in the diabetic control and 14.3% of the rats glomerular focal necrosis was not seen, while 66.7% of the rats in the diabetic control group which received 30 mg pioglitazone group did not manifest any evidence of glomerular focal necrosis. 

 A statistically significant difference was not found as for all histopathological changes between 10 mg and 30 mg pioglitazone groups. In none of these groups, glomerulosclerosis and interstitial fibrosis were observed.

A statistically significant difference was found between the diabetic control and pioglitazone groups regarding tubular dilation, tubular epithelial necrosis, and vascular wall thickening (*P* = 0.023, *P* = 0.034, *P* = 0.005, and *P* = 0.001, resp.) (Table 4).

Rates of severe degrees of vascular wall thickening were 85.7% in the diabetic control, and only 6.7% in the pioglitazone groups. Tubular epithelial necrosis was not observed in 14.3 of the rats in the diabetic control group, while these lesions were not detected in 66.7% of the rats in the pioglitazone groups. Rates of severe degrees of tubular dilation were 71.4% in the diabetic control and only 13.3 in the pioglitazone groups. Still, severe glomerular focal necrosis was seen at a rate of 57.1% in the diabetic control group, while in the pioglitazone groups any evidence of severe glomerular focal necrosis was not encountered.

## 4. Discussion

Current treatment guidelines for diabetic nephropathy recommend achievement of the following targets: systolic blood pressure < 130 mmHg, diastolic blood pressure < 80 mmHg, HbA1c < 7%, and daily protein intake ≤ 0.8 g/kg. Besides, life style modifications such as cessation of smoking, weight loss, regular exercise, restriction of alcohol, and salt are also recommended [24]. Pharmacological treatment modalities with established efficacy in microalbuminuric patients consist of administration of ACE (angiotensin converting enzyme) inhibitors or ARBs (angiotensin converting enzyme receptor blockers) [24, 25].

 In recent years, molecular mechanisms involved in the etiopathogenesis of diabetic nephropathy have been more strongly emphasized in investigations directed at preventive attempts against development of this morbidity. In diabetics, rates of production of free radicals and especially free oxygen radicals in all tissues increase depending on autooxidation of glucose and protein glycation. Besides, a defect develops in cellular defense mechanism against free oxygen radicals. Functions of some antioxidants as CAT, GSH-px, and SOD alter unfavorably [26–28]. Different results have been obtained in studies inquiring diabetes mellitus and the status of antioxidant enzymes. In diabetes- induced rats, free radical scavenger enzymes were measured, and decreases in the activities of glutathione peroxidase, catalase, and superoxide dismutase were observed [29].

Kȩdziora-Kornatowska et al. investigated differences between activities of lipid peroxidation and antioxidant enzymes in 21 proteinuric, 14 normoalbuminuric type 2 patients, and 19 healthy volunteers and detected higher lipid peroxidation activity, but lower antioxidant enzyme (superoxide dismutase and catalase) activity in all patients when compared with the control group. In comparisons among diabetics, these unfavorable results were more marked in patients with proteinuria [30]. Craven et al. reported that when compared with healthy mice in the control group, kidney weight, glomerular volume, urinary albumin output, and glomerular production of TGF-*β*1 had increased in diabetic group, and these unfavorable developments were found to be significantly decreased in the group which received antioxidant vitamin C [31].

 A few studies investigated the effect of pioglitazone on histopathological changes in diabetic nephropathy. In a study performed by Tanimoto et al., volumes of glomeruli and Bowman capsules were measured in rats which received 10 mg/kg pioglitazone therapy. Normalization of the Bowman capsular volume, decrease in the amount of ecNOS (endothelial constitutive nitric oxide synthase) protein in the glomerular vascular endothelium, and improvement in glomerular hyperfiltration were detected after treatment, and intergroup changes in glycemic levels were evaluated as insignificant [32]. Contrarily, in a study performed by Dobrian et al., investigators demonstrated that pioglitazone increased NO expression with resultant decrease in renal oxidative stress [33]. In our study, renal NO levels did not differ between groups with or without pioglitazone therapy. 

Several investigators reported increased levels of MDA (an indicator of lipid peroxidation) in renal tissues of diabetic rats as a result of oxidative stress [34–36]. Also, in our study significant difference was detected in MDA values between the control and the diabetic control groups. Significantly higher levels of MDA were detected in diabetic rats. However, any difference was not detected between the diabetic control and pioglitazone groups.

 In a study performed by Gumieniczek, blood glucose levels were not affected by the pioglitazone therapy in diabetic rats. Also, SOD and CAT levels in renal tissue were measured, and relative to controls, especially, in diabetics SOD was found to be decreased and pioglitazone therapy did not exert any effect on its levels. Higher levels of CAT were detected in diabetic rats which declined significantly in the pioglitazone group. Only diabetic rats given pioglitazone had lower glutathione reductase and glutathione levels in their kidneys. A significant difference in the level of GSHPx was not found [37]. In our study, a statistically significant difference was not found between the diabetic control and pioglitazone groups as for mean SOD, CAT, MDA, and GSH levels. Besides, any blood glucose lowering effect of pioglitazone was not detected. 

Diabetic patients have usually mild degrees of inflammation. TZDs have an anti-inflammatory activity, which is confirmed to occur independently from its hypoglycemic effects in diabetics. Desfaits et al. demonstrated a marked increase in TNF-*α* release from monocytes in type 2 diabetes [38]. Agarwal et al. evaluated inflammatory parameters (IL-6, TNF-*α*) and a lipid oxidation parameter MDA. MDA levels had not changed, and a significant decline was detected in IL-6 levels during posttreatment period in the pioglitazone group. However, TNF-*α* levels had not changed significantly [16]. There was no significant difference for TNF-*α* and IL-6 levels between pioglitazone-treated and the diabetic control groups in our study.

In conclusion, histopathological changes as focal necrosis, tubular epithelial necrosis, tubular dilation, and vascular wall thickening seen in the diabetic group regressed with pioglitazone treatment. This effect of pioglitazone was independent of its antiglycemic activity. However, any difference between groups with and without pioglitazone treatment, as for tissue antioxidant, and inflammatory parameters was not detected. According to this outcome, pioglitazone might prevent the development of diabetic nephropathy; however, larger scale investigations are needed to determine its effect on the antioxidant system.

## Figures and Tables

**Table 1:** Blood glucose level (mg/dL) at the 1st week.

	Control group (*n* = 10)	Diabetic control group (*n* = 10)	10 mg pioglitazone group (*n* = 10)	30 mg pioglitazone group (*n* = 10)
Blood glucose level				
Mean ± SD (min–max)	126 **±** 15.8 (106–162)	557 **±** 71.1 (406–600)	431 **±** 72.1 (320–544)	453 **±** 77.2 (330–538)

**Table 2:** Blood glucose level (mg/dL) at the 4th week.

	Control group (*n* = 10)	Diabetic control group (*n* = 10)	10 mg pioglitazone group (*n* = 10)	30 mg pioglitazone group (*n* = 10)
Blood glucose level				
Mean ± SD (min–max)	131 ± 15.7 (108–154)	464 ± 84.4 (323–579)	459 ± 42.8 (394–511)	387 ± 173.5 (155–530)

**Table 3:** Renal tissue levels of superoxide dismutase, catalase, reduced glutathione, malondialdehyde, nitric oxide, tumor necrosis factor-alpha, and interleukin-6.

	SOD U/mg protein	CAT k/s/mg protein	GSH mg/g protein	MDA nmol/mg protein	NO pg/mg protein	TNF-*α* pg/mg protein	IL-6 ng/g protein
Control group (*n* = 10)	6.1 ± 2.7	1.8 ± 0.6	61.5 ± 41.8	1.3 ± 0.5	51.3 ± 45.5	191.8 ± 73.11	348.7 ± 137.9
Diabetic control group (*n* = 10)	5.1 ± 4.81	1.5 ± 0.6	56.6 ± 31.0	2.8 ± 1.5*	16.6 ± 11.8	183.7 ± 36.8	323.3 ± 93.3
10 mg pioglitazone group (*n* = 10)	6.9 ± 3.1	1.7 ± 0.7	92.3 ± 32.7	2.1 ± 1.1	15.1 ± 8.8	207.8 ± 86.6	329.7 ± 146.7
30 mg pioglitazone group (*n* = 10)	5.7 ± 2.9	2.6 ± 2.2	74.5 ± 47.8	1.7 ± 0.6	22.8 ± 20.8	198.1 ± 68.3	358.2 ± 46.2

SOD: superoxide dismutase; CAT: catalase; GSHr: reduced glutathione; MDA: malondialdehyde; NO: nitric oxide; TNF-*α*: tumor necrosis factor-alpha; IL-6: Interleukin-6. *P < 0.05.

**Table 4:** Histopathological changes in diabetic control and pioglitazone groups.

	Diabetic control			Pioglitazone groups		
	− (%)	+ (%)	++ (%)	− (%)	+ (%)	++ (%)
Vascular wall thickening*	1 (14.3%)	0 (0%)	6 (85.7%)	6 (40%)	8 (53.3%)	1 (6.7%)
Tubular epithelial necrosis*	1 (14.3%)	6 (85.7%)	0 (0.0%)	10 (66.7%)	4 (26.7%)	1 (6.7%)
Tubular dilation*	0 (0.0%)	2 (28.6%)	5 (71.4%)	1 (6.7%)	12 (80%)	2 (13.3%)
Glomerular focal necrosis	1 (14.3%)	2 (28.6%)	4 (57.1%)	8 (53.3%)	7 (46.7%)	0 (0.0%)
Dilation of Bowman capsule	0 (0.0%)	6 (85.7%)	1 (14.3%)	5 (33%)	8 (53.3%)	2 (13.3%)
Congestion	0 (0.0%)	7 (100%)	0 (28.6%)	0 (0.0%)	12 (80%)	3 (20%)
Degeneration of tubular epithelium	0 (0.0%)	5 (71.4%)	2 (28.6%)	3 (20%)	12 (80%)	0 (0.0%)
Interstitial inflammation	4 (57.1%)	3 (42.9%)	0 (0.0%)	8 (53.3%)	6 (40%)	1 (6.7%)

(−) unaffected, (+) moderately affected, (++) severe affected. *P < 0.05.
